# Dr. George W. Keely

**Published:** 1888-09

**Authors:** 


### Obituary.

Dr George W. Keely Dead.—Hosts of our readers will receive a shock when they read this announcement.

Dr. Keely was enjoying his usual perfect health until Wednesday evening (August 22), when the accident befell him which caused his death the following Friday afternoon at two o'clock (August 24, 1888). On the evening of the 22d the doctor went up into the third story of the building in Oxford, Ohio, in which his office is situated, to mend the wire of a private telephone connecting his office and residence. The wire could be easily reached with a hoe from a rear window. The doctor sat sideways in the window, with a hoe in one hand and a pocket pen-knife in the other, when, from dizziness or some cause, he fell from the window into a court, some twenty-eight feet below. He must have remained unconscious for two or three hours, when, recovering his senses, he, with great difficulty, was able to crawl to the front of the building on the street, where, in time, he was found by Marshal Kyler, who was patrolling the town. This was about midnight. The d c-tor was helped to his home, covered with blood and bruises, and, as was found by the surgeon, Dr. Hill, of Oxford, who was immediately summoned, the broken-bladed pen-knife sticking firmly in the skull. This was removed only after considerable difficulty and after the operation of trephining had been performed. The other injuries that were apparent after examination were some bad fractures of the fingers of the left hand and bruises of the head, back and shoulders. Dr. Keely, however, seemed so comfortable at five o'clock on the morning after the accident that Dr. Hill felt secure in leaving him and coming to the city for a couple of days. On Friday morning it was found that some of the ribs had been fractured, causing internal injury, from which the doctor rapidly sank until two o'clock, when he died.

Dr. Keely was in his 65th year, and though old in years was as vigorous in health and intellect as ever He was as enthusiastic in his profession, as careful a student, as thorough an experimentor, as anyone could be in the beginning of a professional career. His name is known wherever "American Dentistry" is known, any where in the world. He has held the highest places in the dental societies of which he has always been an active member. At the time of his death he held the office of Treasurer of the American Dental Association, which met in Louisville, Ky., and the doctor had prepared to attend that meeting.

Born in Oxford, Ohio. and keeping his residence there all his life within a stone's throw of the old homestead, the doctor has become so identified with the town for half a century that no history of Oxford could be complete without constant reference to the acts of kindness and usefulness and unselfish work on the part of Dr. Keely for the benefit of his native village. He was a trustee of the Ohio Dental College of Cincinnati, and always active in its councils. He was a lecturer in the college on his special branch, and has held many hundreds of students deeply interested in his lectures. A good man, a useful man, a kind and genial man, a man whom everybody loved, has gone. We shall not meet his like again.

The town of Oxford and the societies and institutions of which the doctor was a member will, in due time, memorialize his sad taking away. C. M. W.

—*Cincinnati Medical Journal.*

---

